# Supplementary material for: The NMDA receptor antagonist ketamine impairs and delays context-dependent decision making in the parietal cortex
Source: Commun Biol. 2022 Jul 20;5:690. doi: 10.1038/s42003-022-03626-z (PMC9300646; doi:10.1038/s42003-022-03626-z)
Supplement: Supplementary file 2 — Supplementary Information [file 42003_2022_3626_MOESM2_ESM.pdf]

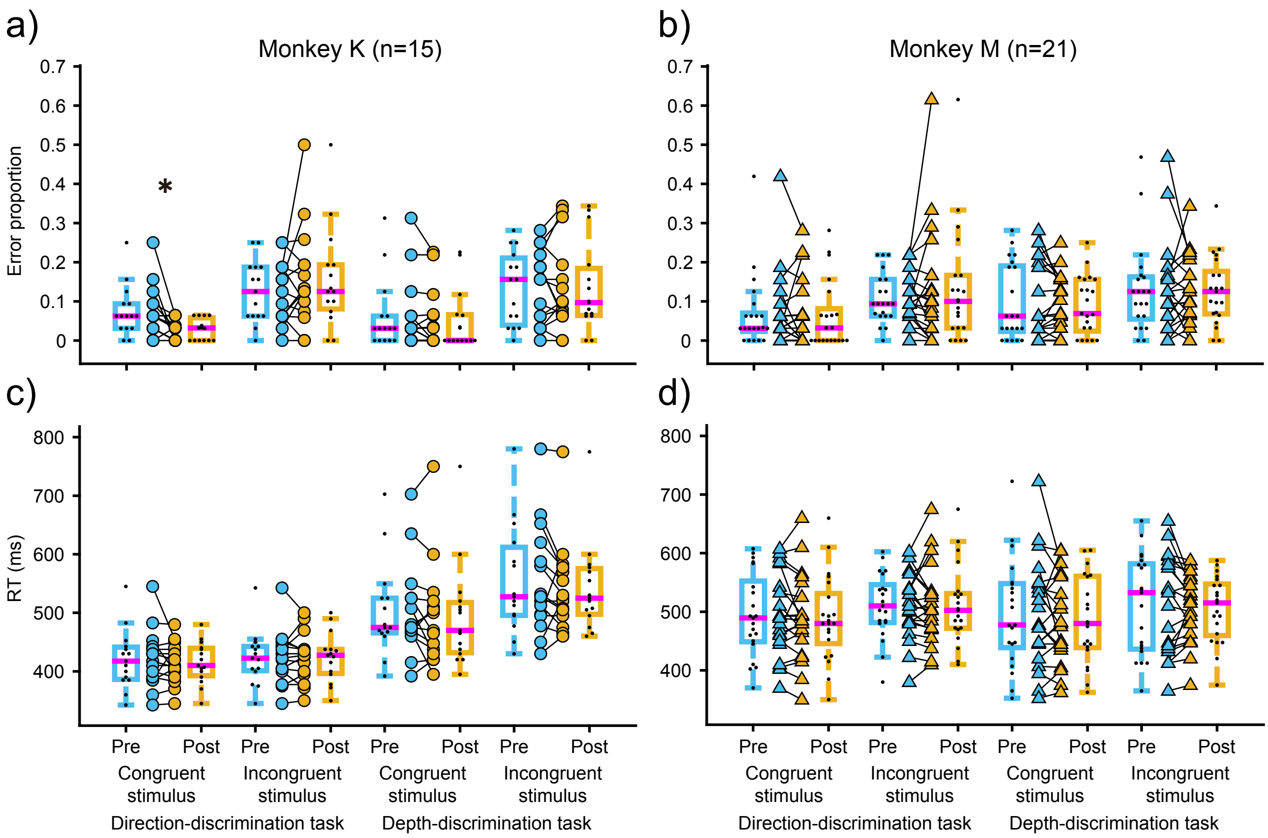

Supplementary Figure 1. Effects of saline on task-switching performance for stimulus congruency.

Boxplots show the error proportion (a, b) and reaction time (c, d) for congruent and incongruent stimuli with saline administration for the direction-discrimination task and the depth-discrimination task. Boxplot and other conventions are the same as in Figure 2.

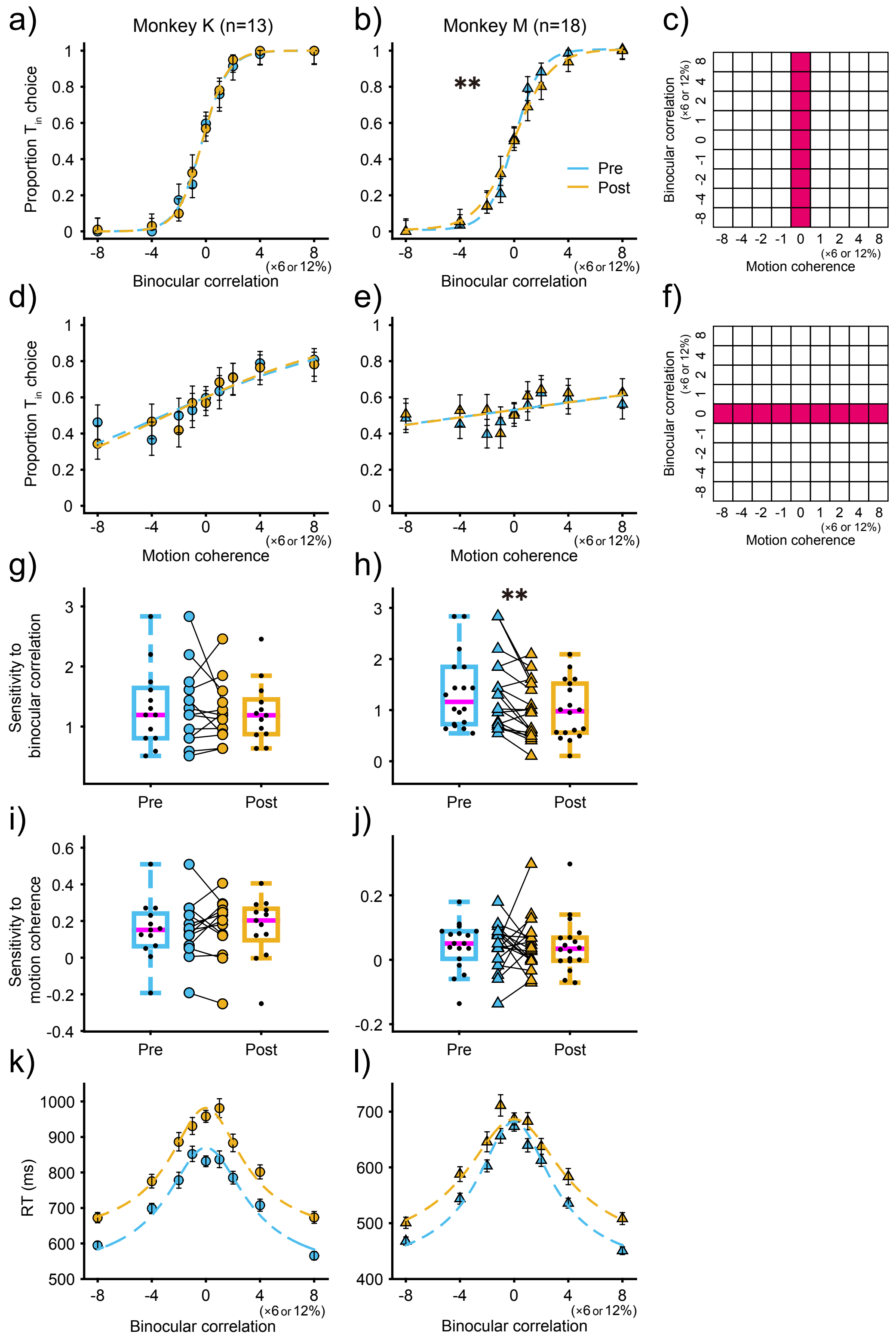

Supplementary Figure 2. Effects of ketamine on choice and reaction time for the depth-discrimination task.

a-c) Psychometric function before (blue) and after (yellow) ketamine administration for each monkey. Proportion Tin choice is plotted as a function of binocular correlation with 0% motion coherence. The conventions are the same as in Figure 3. d-f) Proportion Tin choice is plotted as a function of motion coherence. g-h) Ketamine effect on behavioral sensitivity to binocular correlation. i-j) Ketamine effect on behavioral sensitivity to motion coherence. k-l) Change in chronometric function with ketamine administration. RTs are plotted as a function of binocular correlation.

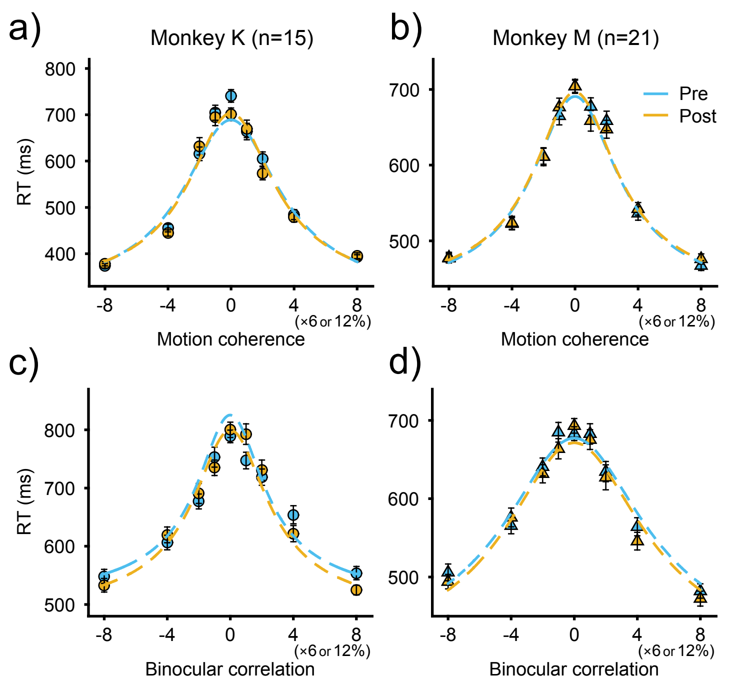

Supplementary Figure 3. Effects of saline on reaction time for both tasks. RTs for monkey K (a, c) and monkey M (b, d) are plotted for the direction-discrimination task (a, b) and the depth-discrimination task (c, d). The conventions are the same as in Figure 3k-l.

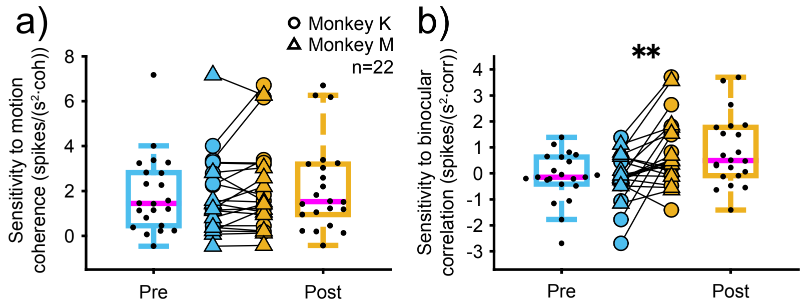

Supplementary Figure 4. LIP sensitivity calculated with a time window identical for pre- and post-ketamine administration.

LIP sensitivity to motion coherence (a) and binocular correlation (b) derived from build-up slope are plotted for the direction-discrimination task before and after ketamine administration. The build-up slope was calculated by using data from 200 ms to 400 ms after visual stimulus onset. Asterisks indicate statistically significant results (Wilcoxon signed-rank test; $^{**}p < 0.01$). Boxplot and other conventions are the same as in Figure 4e-f.

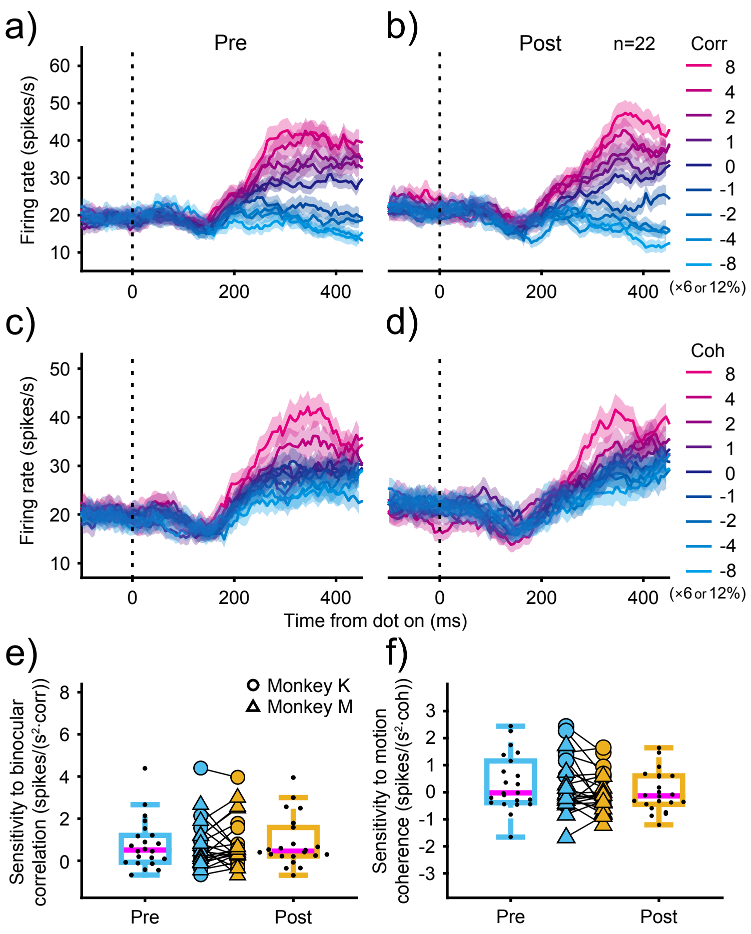

Supplementary Figure 5. Effects of ketamine on average LIP responses for the depth-discrimination task.

a, b) Average firing rates across 22 LIP neurons are plotted before (left) and after (right) ketamine administration. Each colored line denotes average firing rates at different binocular correlation with motion coherence fixed at zero. Dotted-vertical line denotes the time of visual stimulus onset. Shaded area denotes SEM. c, d) Average LIP responses for different motion coherences. The conventions are the same as in (a, b). e, f) LIP sensitivity derived from the build-up slope before and after administration. Boxplots show the effects of ketamine on the sensitivity to binocular correlation (e) and motion coherence (f) for the depth-discrimination task. Circles and triangles denote each experimental data for monkey K and monkey M, respectively. Boxplot conventions are the same as in Figure 2.

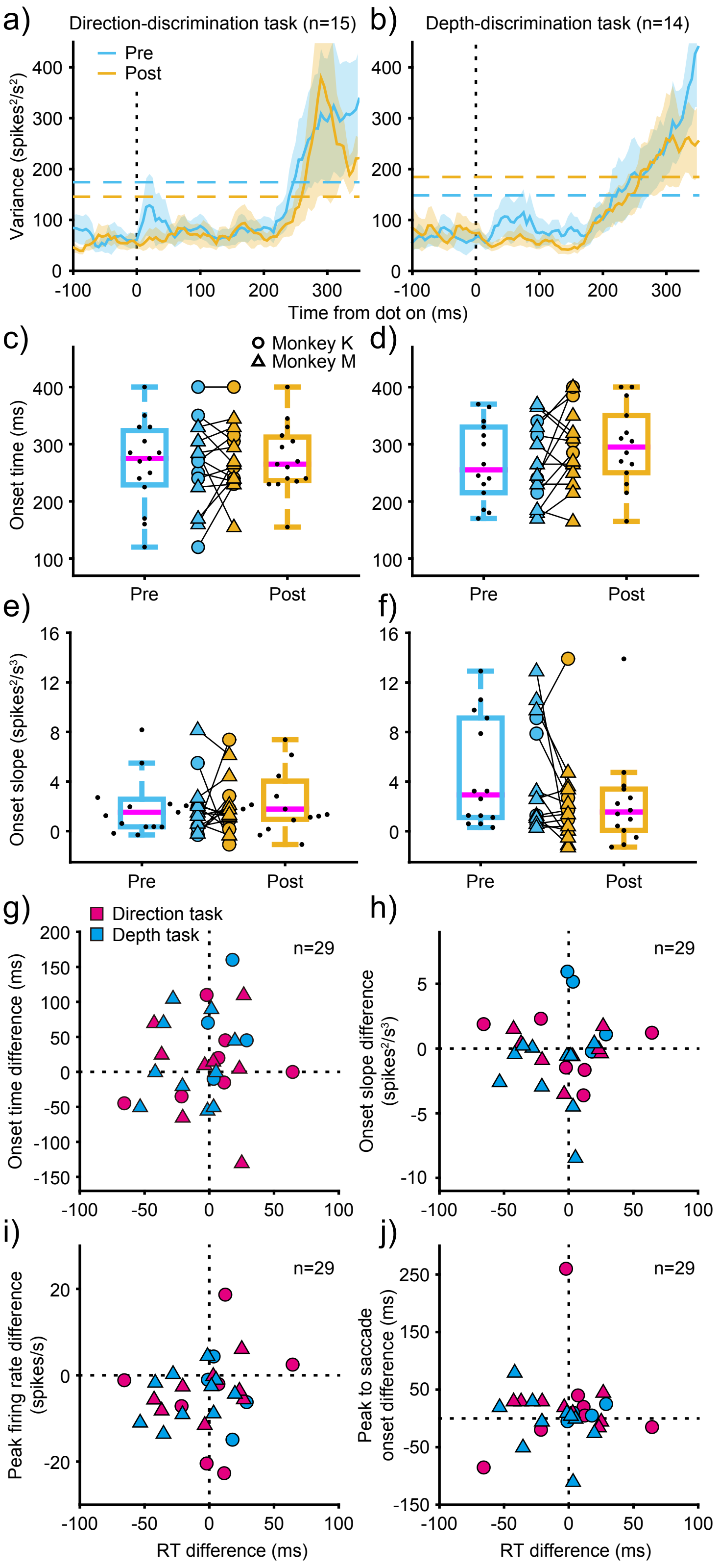

Supplementary Figure 6. Effects of saline on build-up onset.
a, b) Build-up onset of LIP neurons before and after saline administration for the direction-discrimination task (a) and the depth-discrimination task (b). The conventions are the same as in Figure 5a-b. c-f) Build-up onset time (c, d) and slope (e, f) before and after saline administration for the direction-discrimination task (c, e) and the depth-discrimination task (d, f). The conventions are the same as in Figure 5c-f. g-j) Relationship between RT delay and build-up onset delay (g), build-up onset slope difference (h), peak firing rate difference (i) and peak to saccade onset difference (j). The conventions are the same as in Figure 5g-j.

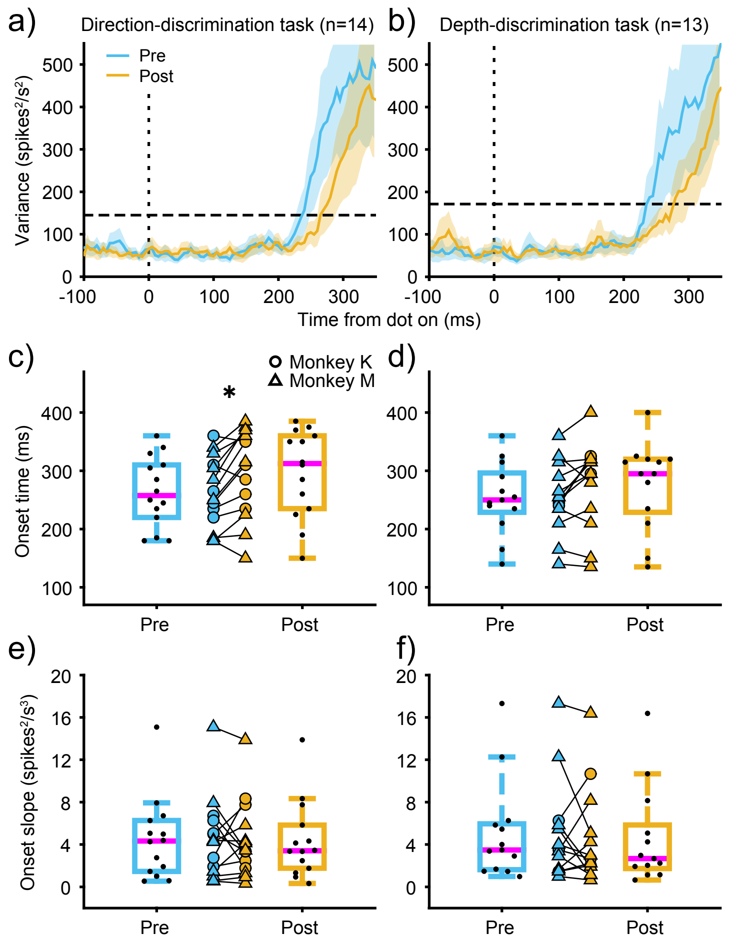

Supplementary Figure 7. Effects of ketamine on build-up onset determined from a single threshold.

a, b) Build-up onset of LIP neurons before and after ketamine administration for the direction-discrimination task (a) and the depth-discrimination task (b) determined from a single threshold. The threshold denoted with a dashed-horizontal line was calculated from the baseline period of both the pre- and post- conditions. Other conventions are the same as in figure 5a-b. c-f) Build-up onset time (c, d) and slope (e, f) before and after ketamine administration for the direction-discrimination task (c, e) and the depth-discrimination task (d, f) determined from a single threshold. Asterisks indicate the statistically significant results (Wilcoxon signed-rank test; *$p < 0.05$). Other conventions are the same as in Figure 5c-f.

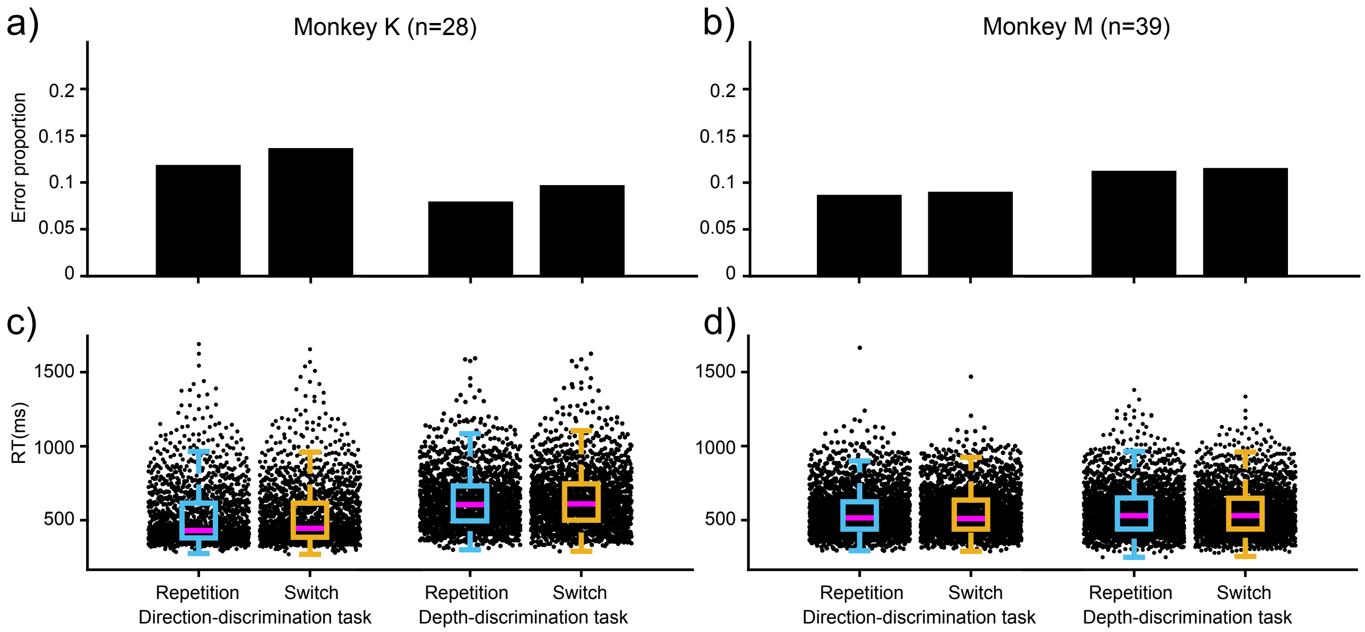

Supplementary Figure 8. Task-switching performance between repetition and switch trials.

Error proportion (a, b) and reaction time (c, d) for repetition and switch trials are plotted for monkey K (a, c) and monkey M (b, d), respectively. Data involve pre-administration trials from both saline (n = 36) and ketamine (n = 31) experiments. Small dots denote data from each trial. Boxplot conventions are the same as in Figure 2. There were no significant differences between the repetition and switch trials ($p > 0.05$, Chi-square test for (a, b), Wilcoxon rank sum test for (c, d), Supplementary Table 5).

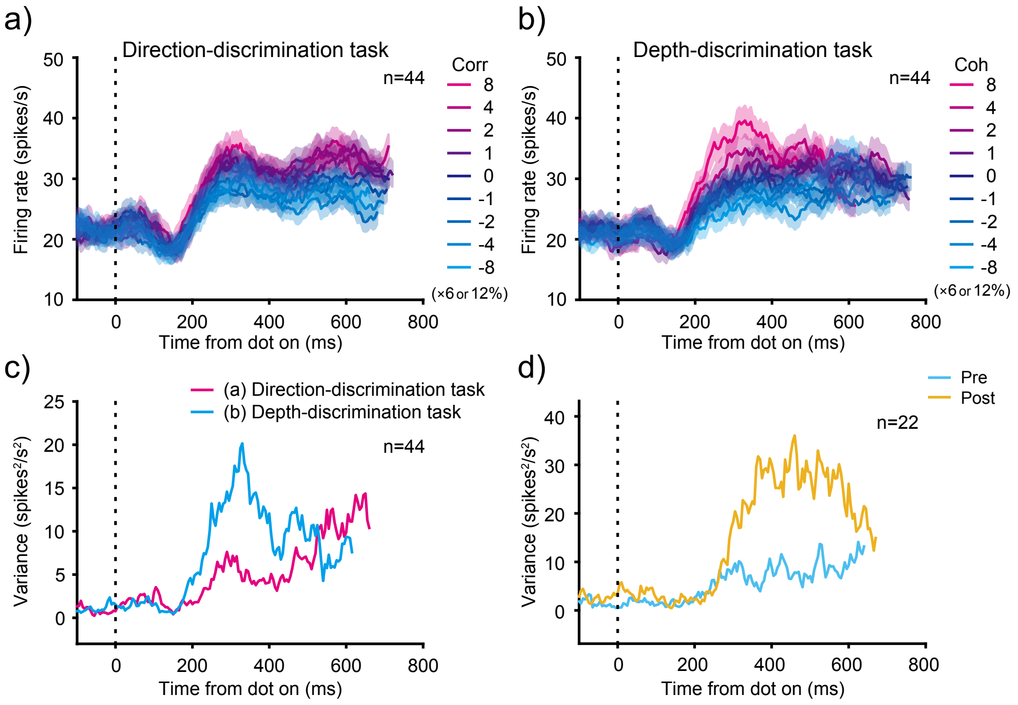

Supplementary Figure 9. Task-dependent differences in LIP build-up.
a, b) Average firing rate among 44 LIP neurons. Colored lines indicate responses to different binocular correlations in the direction-discrimination task (a), and different motion coherences in the depth-discrimination task (b). Firing rates were calculated from pre-administration trials for both saline (n = 22) and ketamine (n = 22) experiments. Dotted-vertical line denotes the time of visual stimulus onset. Shaded area denotes SEM. c) Comparison of firing rate variance. Variances among stimulus conditions are plotted in magenta for (a) and in cyan for (b). d) Ketamine effect on firing rate variance. Variance among different binocular correlations for the direction-discrimination task is plotted before (blue) and after (yellow) ketamine administration. Variances were calculated by using data from ketamine experiments (n = 22) recorded from area LIP.

Supplementary Table 1. Summary of behavioral performance for congruent and incongruent stimuli with ketamine administration.

Error proportion

Subject	Task	Stimulus congruency	Pre	Post	p value
Monkey K	Direction-discrimination task	Congruent	0.094 (0.055, 0.13)	0.063 (0.024, 0.065)	0.11
		Incongruent	0.094 (0.094, 0.16)	0.22 (0.12, 0.31)	0.040
	Depth-discrimination task	Congruent	0.031 (0.023, 0.070)	0.032 (0, 0.078)	0.58
		Incongruent	0.19 (0.063, 0.19)	0.19 (0.15, 0.24)	0.41
Monkey M	Direction-discrimination task	Congruent	0.031 (0.031, 0.063)	0.041 (0, 0.093)	0.30
		Incongruent	0.078 (0.031, 0.19)	0.13 (0.067, 0.22)	0.031
	Depth-discrimination task	Congruent	0.047 (0, 0.13)	0.056 (0, 0.13)	0.90
		Incongruent	0.094 (0.031, 0.13)	0.098 (0.045, 0.18)	0.078

Reaction time (ms)

Subject	Task	Stimulus congruency	Pre	Post	p value
Monkey K	Direction-discrimination task	Congruent	410 (390, 453)	470 (439, 514)	0.0015
		Incongruent	413 (391, 435)	465 (449, 527)	<0.001
	Depth-discrimination task	Congruent	545 (500, 572)	595 (533, 638)	0.0049
		Incongruent	590 (558, 623)	665 (640, 710)	0.0017
Monkey M	Direction-discrimination task	Congruent	459 (420, 520)	499 (463, 525)	0.012
		Incongruent	474 (430, 540)	518 (485, 553)	<0.001
	Depth-discrimination task	Congruent	468 (430, 503)	490 (478, 525)	0.0074
		Incongruent	491 (455, 525)	511 (480, 563)	0.044

Each value denotes median (interquartile range: Q1, Q3) across experiments. P values were calculated using the Wilcoxon signed-rank test.

Supplementary Table 2. Model comparisons using Akaike’s Information Criterion (AIC)

Subject	Drug	Full model	Excluded parameter		
			dk	da	dt_R
Monkey K	Ketamine	325	321	321	342
	Saline	393	390	389	400
Monkey M	Ketamine	274	280	274	306
	Saline	335	331	331	332

Each value denotes Akaike information criterion of the full model, and models that excluded each difference parameter in the drift diffusion model.

Supplementary Table 3. Summary of build-up onset, variance slope, peak firing rate, and peak to saccade onset of LIP neurons.

	Task	Drug	Pre	Post	p value
Build-up onset (ms)	Direction-discrimination task	Ketamine	257.5 (225.0, 300.0)	312.5 (247.5, 350.0)	0.002
		Saline	275.0 (228.8, 323.8)	265.0 (236.3, 312.5)	0.572
	Depth-discrimination task	Ketamine	250.0 (213.8, 308.8)	310.0 (237.5, 330.0)	0.066
		Saline	255.0 (215.0, 330.0)	295.0 (250.0, 350.0)	0.172
Variance slope at build-up onset (spikes^2/s^3)	Direction-discrimination task	Ketamine	3.50 (0.86, 5.66)	3.35 (1.52, 6.79)	0.642
		Saline	1.70 (1.34, 4.72)	2.29 (1.08, 3.93)	0.804
	Depth-discrimination task	Ketamine	3.38 (1.48, 4.87)	4.04 (1.91, 8.10)	0.524
		Saline	1.96 (1.16, 6.54)	2.19 (0.68, 2.93)	0.502
Peak firing rate (spikes/s)	Direction-discrimination task	Ketamine	73.9 (43.2, 87.0)	68.9 (53.7, 105.2)	0.877
		Saline	63.2 (47.8, 86.7)	62.5 (43.1, 80.3)	0.055
	Depth-discrimination task	Ketamine	74.3 (42.4, 92.7)	60.6 (47.1, 112.5)	0.252
		Saline	65.7 (51.2, 92.6)	61.3 (47.1, 80.0)	0.020
Time from build-up peak to saccade onset (ms)	Direction-discrimination task	Ketamine	-47.5 (-72.5, 22.5)	-35.0 (-77.5, 60.0)	0.894
		Saline	-15.0 (-97.5, 23.8)	0 (-78.8, 43.8)	0.111
	Depth-discrimination task	Ketamine	-65.0 (-97.5, 40.0)	-65.0 (-136.3, 32.5)	0.145
		Saline	-22.5 (-85.0, 35.0)	-52.5 (-75.0, 20.0)	0.598

Each value denotes median (interquartile range: Q1, Q3) across neurons. P values were calculated using the Wilcoxon signed-rank test.

Supplementary Table 4. Summary of neural sensitivity and response onset of MT neurons.

		Drug	Pre	Post	p value
Neural sensitivity (spikes/(s・corr))	Preferred stimulus	Ketamine	0.12 (0.05, 0.25)	0.10 (0.04, 0.29)	0.73
		Saline	0.12 (0.02, 0.20)	0.12 (0.02, 0.20)	0.58
	Anti-preferred stimulus	Ketamine	-0.16 (-0.20, 0)	-0.14 (-0.22, -0.06)	0.25
		Saline	-0.01 (-0.09, 0)	-0.03 (-0.08, 0)	1.00
Response onset (ms)		Ketamine	67.5 (40.0, 92.5)	70.0 (40.0, 110.0)	1.00
		Saline	107.5 (80.0, 115.0)	102.5 (60.0, 120.0)	0.48

Each value denotes median (interquartile range: Q1, Q3) across neurons. P values were calculated using the Wilcoxon signed-rank test.

Supplementary Table 5. Summary of behavioral performance between repetition and switch trial.

Subject	Task		Repetition	Switch	p value
Monkey K	Direction-discrimination task	Error proportion	0.12	0.14	0.11
		Reaction time (ms)	430 (380, 615)	445 (385, 615)	0.055
	Depth-discrimination task	Error proportion	0.078	0.096	0.063
		Reaction time (ms)	605 (495, 731)	610 (500, 745)	0.35
Monkey M	Direction-discrimination task	Error proportion	0.085	0.089	0.67
		Reaction time (ms)	515 (440, 625)	510 (440, 636)	0.94
	Depth-discrimination task	Error proportion	0.11	0.11	0.75
		Reaction time (ms)	530 (440, 650)	530 (440, 648)	0.76

Error proportion value denotes error ratio, and reaction time value denotes median (interquartile range: Q1, Q3) of all trial data. P values were calculated using chi-square test for error proportion, and Wilcoxon signed-rank test for reaction time.
