# Supplementary material for: The NMDA receptor antagonist ketamine impairs and delays context-dependent decision making in the parietal cortex
Source: Commun Biol. 2022 Jul 20;5:690. doi: 10.1038/s42003-022-03626-z (PMC9300646; doi:10.1038/s42003-022-03626-z)
Supplement: Supplementary file 3 — Description of Additional Supplementary Files [file 42003_2022_3626_MOESM3_ESM.pdf]

Description of Additional Supplementary Files

File Name: Supplementary Data 1

Description: The source data behind the figure 2

File Name: Supplementary Data 2

Description: The source data behind the figure 3

File Name: Supplementary Data 3
Description: The source data behind the figure 4e-f

File Name: Supplementary Data 4

Description: The source data behind the figure 5c-f

File Name: Supplementary Data 5

Description: The source data behind the figure 6c,d,f

File Name: Supplementary Data 6

Description: The source data behind the figure 7
