# Supplementary material for: The NMDA receptor antagonist ketamine impairs and delays context-dependent decision making in the parietal cortex
Source: Commun Biol. 2022 Jul 20;5:690. doi: 10.1038/s42003-022-03626-z (PMC9300646; doi:10.1038/s42003-022-03626-z)
Supplement: Supplementary file 10 — Reporting Summary [file 42003_2022_3626_MOESM10_ESM.pdf]

nature research

Corresponding author(s): Takanori Uka

Last updated by author(s): Jun 7, 2022

Reporting Summary

Nature Research wishes to improve the reproducibility of the work that we publish. This form provides structure for consistency and transparency in reporting. For further information on Nature Research policies, see our Editorial Policies and the Editorial Policy Checklist.

Statistics

For all statistical analyses, confirm that the following items are present in the figure legend, table legend, main text, or Methods section.

n/a	Confirmed	
☐	☒	The exact sample size (n) for each experimental group/condition, given as a discrete number and unit of measurement
☐	☒	A statement on whether measurements were taken from distinct samples or whether the same sample was measured repeatedly
☐	☒	The statistical test(s) used AND whether they are one- or two-sided<br>*Only common tests should be described solely by name; describe more complex techniques in the Methods section.*
☐	☒	A description of all covariates tested
☐	☒	A description of any assumptions or corrections, such as tests of normality and adjustment for multiple comparisons
☐	☒	A full description of the statistical parameters including central tendency (e.g. means) or other basic estimates (e.g. regression coefficient) AND variation (e.g. standard deviation) or associated estimates of uncertainty (e.g. confidence intervals)
☐	☒	For null hypothesis testing, the test statistic (e.g. F, t, r) with confidence intervals, effect sizes, degrees of freedom and P value noted<br>*Give P values as exact values whenever suitable.*
☐	☒	For Bayesian analysis, information on the choice of priors and Markov chain Monte Carlo settings
☐	☒	For hierarchical and complex designs, identification of the appropriate level for tests and full reporting of outcomes
☐	☒	Estimates of effect sizes (e.g. Cohen's d, Pearson's r), indicating how they were calculated

Our web collection on statistics for biologists contains articles on many of the points above.

Software and code

Policy information about availability of computer code

Data collection	We used a commercially available software package (TEMPO, Reflective Computing) for controlling behavioral tasks and data acquisition. We recorded extracellular activity from isolated neurons using an online spike sorting software (OmniPlex Software, PLEXON Inc.).
Data analysis	All data analyses were performed using custom code written in MATLAB (The MathWorks).

For manuscripts utilizing custom algorithms or software that are central to the research but not yet described in published literature, software must be made available to editors and reviewers. We strongly encourage code deposition in a community repository (e.g. GitHub). See the Nature Research guidelines for submitting code & software for further information.

Data

Policy information about availability of data

All manuscripts must include a data availability statement. This statement should provide the following information, where applicable:

- Accession codes, unique identifiers, or web links for publicly available datasets
- A list of figures that have associated raw data
- A description of any restrictions on data availability

The data that support the findings of this study will be available from the corresponding author upon request.

Field-specific reporting

Please select the one below that is the best fit for your research. If you are not sure, read the appropriate sections before making your selection.

☒ Life sciences ☐ Behavioural & social sciences ☐ Ecological, evolutionary & environmental sciences

For a reference copy of the document with all sections, see nature.com/documents/nr-reporting-summary-flat.pdf

Life sciences study design

All studies must disclose on these points even when the disclosure is negative.

Sample size	Although we didn't determine sample size before experiments, we confirmed sufficient statistical difference between before and after drug administration.
Data exclusions	We didn't exclude any data of experiments.
Replication	We recorded data from two monkeys, and conducted multiple experiments in each monkey.
Randomization	Stimuli were presented pseudorandomly. Saline and ketamine was administered in a random order.
Blinding	Data collection and analysis were not performed blind to the conditions of the experiments. However, we did not select the type of neurons during neural data acquisition.

Reporting for specific materials, systems and methods

We require information from authors about some types of materials, experimental systems and methods used in many studies. Here, indicate whether each material, system or method listed is relevant to your study. If you are not sure if a list item applies to your research, read the appropriate section before selecting a response.

Materials & experimental systems

n/a	Involved in the study	
☒	☐	Antibodies
☒	☐	Eukaryotic cell lines
☒	☐	Palaeontology and archaeology
☐	☒	Animals and other organisms
☒	☐	Human research participants
☒	☐	Clinical data
☒	☐	Dual use research of concern

Methods

n/a	Involved in the study	
☒	☐	ChIP-seq
☒	☐	Flow cytometry
☒	☐	MRI-based neuroimaging

Animals and other organisms

Policy information about studies involving animals; ARRIVE guidelines recommended for reporting animal research

Laboratory animals	We used two male Japanese monkeys (Macaca fuscata). One is approximately 10-year-old, and the other is older than 12-year-old.
Wild animals	We did not use wild animals.
Field-collected samples	We did not use wild animals.
Ethics oversight	The animal care and experimental procedures were approved by the Animal Care and Use Committees of Juntendo University, Tamagawa University, and the University of Yamanashi in accordance with NIH guidelines.

Note that full information on the approval of the study protocol must also be provided in the manuscript.
